# Environmental, Socioeconomic, Maternal, and Breastfeeding Factors Associated with Childhood Overweight and Obesity in Ceará, Brazil: A Population-Based Study

**DOI:** 10.3390/ijerph17051557

**Published:** 2020-02-28

**Authors:** Sabrina G. M. O. Rocha, Hermano A. L. Rocha, Álvaro J. M. Leite, Márcia M. T. Machado, Ana C. Lindsay, Jocileide S. Campos, Antônio J. L. A. Cunha, Anamaria C. e Silva, Luciano L. Correia

**Affiliations:** 1 Department of Community Health, Federal University of Ceará, Fortaleza 60430-23, Brazil; sabrinagmor@gmail.com (S.G.M.O.R.); marciamachadoufc@gmail.com (M.M.T.M.); correialuciano@hotmail.com (L.L.C.); 2 Integração, Serviço, Ensino e Comunidade, University Center Unichristus, Fortaleza 60190-180, Brazil; jocileide23@gmail.com (J.S.C.); anamariacs2013@gmail.com (A.C.e.S.); 3 Department of Global Health and Population, Harvard T. H. Chan School of Public Health, Boston, MA 02115, USA; 4 Department of Maternal and Child Health, Federal University of Ceará, Fortaleza, 60430140, Brazil; alvaromadeiro@yahoo.com.br; 5 Department of Exercise and Health Sciences, College of Nursing and Health Sciences, University of Massachusetts, Boston, MA 02125, USA; Ana.Lindsay@umb.edu; 6 Department of Pediatrics, Federal University of Rio de Janeiro, Rio de Janeiro 21941-912, Brazil; antonioledo@yahoo.com.br

**Keywords:** childhood overweight, childhood obesity, determinants, breastfeeding, nutrition, Brazil

## Abstract

Childhood obesity is now an epidemic in many countries worldwide and is known to be a multifactorial condition. We aimed to examine the relationship of environmental, socioeconomic, and nutritional factors with childhood overweight and obesity. We conducted a population-based cross-sectional study of children from 2 to 6 years of age in Ceará, Brazil. Children’s nutritional status was assessed by body mass index (BMI) Z scores categorized as overweight and obesity. Ordinal logistic regression models were used to assess the relationship between the factors with overweight and obesity. A total of 2059 children participated, of which 50.4% were male. The mean age was 46 ± 17 months, with a prevalence of overweight and obesity of 12.0% (95% CI 10.7–13.6) and 8.0% (6.7–9.5), respectively. In multivariate analysis, the probability of childhood obesity increased as family income increased (adjusted hazard ratio (aHR) 0.6 (95% CI 0.37–0.95), *p*-value = 0.03). Moreover, families with fewer children had more than 30% fewer overweight children (aHR 0.68; 95% CI 0.48–0.96). Environmental, socioeconomic, and child nutritional factors were associated with overweight and obesity. The results provided could be used to design integrated interventions spanning from conception, or earlier, through the first years of life and may improve child nutritional outcomes.

## 1. Introduction

The childhood obesity epidemic is finding increasing numbers in many countries worldwide. According to World Health Organization (WHO) estimates, the world had 40.1 million children under five with overweight in 2018. It is estimated that in Africa, the prevalence of 8.5% in 2010 increases to 12.7% in 2020, and by 2025 there will be 70 million overweight or obese children and adolescents, most living in low- and middle-income countries [1].

The increasing incidence of childhood obesity has been attributed to changes in the dietary pattern of the population, resulting from changes in the economic pattern and, consequently, of household consumption [2]. It is a multifactorial condition, determined by genetic, behavioral, environmental, and cultural factors. The main factors associated with childhood obesity in Brazil are overweight of at least one parent, birth weight, socioeconomic status, maternal education, and time of breastfeeding [3]. 

Overweight at an early age increases the risk for various childhood conditions, such as endocrine-metabolic diseases [4], and for comorbidities that may perpetuate into adulthood, such as metabolic syndrome [5,6]. Other comorbidities are identified as consequences of childhood obesity, such as kidney, orthopedic, neurological, and psychosocial problems [7]. These are consequences that increase health spending. In Brazil, it is estimated that the annual spending on obesity is around 2.4% of the country’s gross domestic product (GDP), being the third public health problem that most demands spending of the Brazilian economy, ahead of smoking. Worldwide, 2.8% of all wealth is spent on tackling obesity—as much as the cost of smoking and war [8].

Brazilian children, especially from the semiarid region, have shown increasing numbers of overweight and obesity, while still presenting a high prevalence of malnutrition, demonstrating a double burden scenario of malnutrition in the country [2], that underscores the importance of knowing the factors associated with childhood overweight to guide the elaboration of public policies to combat this condition.

We examined the relationship of environmental, socioeconomic, and nutritional factors with childhood overweight and obesity, through a cross-sectional study of children 2–6 years of age residing in Ceará, Brazil. These data are intended to identify priority risk factors for child and maternal health and to inform high-impact population-based interventions.

## 2. Materials and Methods 

### 2.1. Study Design and Population

We analyzed data from the Pesquisa de Saúde Materno Infantil no Ceará (PESMIC) study and full details of the methods of the parent study and the anthropometric assessment can be found elsewhere [9,10]. Briefly, the PESMIC is a population-based cross-sectional study on maternal and child health of preschool children up to 72 months of age living in the state of Ceará, in northeastern Brazil. Ceará is one of the poorest states in Brazil, with a population of 9 million inhabitants living in a semiarid climate. Fortaleza (2.3 million inhabitants) is the capital and urban commercial center of Ceará, but the study area also includes rural areas of Ceará, where subsistence farming is dominant. 

PESMIC surveys were conducted in 1987, 1990, 1994, 2001, 2007, and 2017 using the same methods. For this analysis, we used child anthropometric data from the 2017 PESMIC survey. The PESMICs used cluster sampling, based on the Brazilian Institute of Geography and Statistics (IBGE) census tracts, and stratification between the state capital Fortaleza and the rural areas. The 2017 PESMIC was conducted from August to November 2017 and surveyed 160 randomly selected census tracts that included a total of 3200 households. In this study, all children from 24 to 72 months old and women 10–50 years old living in selected households were included. To ensure that the study population was representative, municipalities, census tracts, and households were randomly selected. Once a census tract was defined and its corresponding map obtained, the location of the cluster of 20 houses to be investigated was determined as follows: the starting point of the cluster (the first home to be visited) was randomly selected utilizing ArcGIS^®^ software, GIS Inc. Households were visited consecutively, in a counterclockwise spiral fashion. Shops and abandoned buildings were excluded and replaced; and in the case of absent families, up to three return visits were conducted in an attempt to obtain data. In each household, information was obtained about all children through the mother’s or primary caregiver’s report, and child anthropometric measurements were taken by trained staff. Children that were hospitalized during the household visit were excluded from the research.

### 2.2. Overweight and Obesity Assessment

Child overweight and obesity were identified using the WHO definitions, following different standard according to the age of the children [11]:

- WHO Child Growth Standards (from 2 years to age 5) [12]
  Obese: Body mass index (BMI) >3 standard deviations above the WHO growth standard median
  Overweight: BMI > 2 standard deviations above the WHO growth standard median
- WHO Reference 2007 (ages 5–6) [13]
  Obese: Body mass index (BMI) >2 standard deviations above the WHO growth standard median
  Overweight: BMI > 1 standard deviation above the WHO growth standard median

The length of children was measured to the nearest 0.1 cm with the use of a portable stadiometer. Weight was measured to the nearest 0.1 kg with the use of a SECA^®^ digital scale. BMI-for-age Z scores were calculated with the use of WHO child growth standards [12,13]. 

### 2.3. Environmental, Socioeconomic, and Nutritional Factors

Standardized questionnaires were administered to the mother or the person with a larger time with the child of the household. 

We collected data on multiple indicators of socioeconomic status, that were interpreted as predictors of the main outcome. Food insecurity was assessed through the application of the United States Department of Agriculture (USDA) questionnaire modified by the experience of use in Brazil [14]. The instrument consists of 15 central closed questions, with a yes/no response on the experience in the last three months of food insufficiency at its various levels of intensity, ranging from the apprehension that food may be lacking until the experience of passing all day without eating. Each affirmative answer of the questionnaire is equivalent to one point, varying the score from 0 to 15 points, considering the value zero as the safety condition; 1–5 points as mild insecurity; 6–10 points as moderate insecurity, and 11–15 points as severe insecurity. We then dichotomized the variable, using as categories no food insecurity and any food insecurity. Head (or the child-caregiver) of households were asked to report their monthly income in Reais (Brazilian currency) and participation status in Bolsa Família. Social class was determined by the Brazilian Criteria “Critério Brasil”, which was developed using a representative sample of the family budget research (POF), carried out by the IBGE, classifying 55,970 domiciles based on household assets [15]. We categorized this variable in terciles.

Parental education was measured by the number of years of formal education. In Brazil, the first four years of school are basic education; from five to eight years the second basic degree; and more than eight, medium and higher education.

Environmental, maternal morbidities, ethnicity, and breastfeeding retrospective data were reported by the mother and were confirmed with the child health booklet held by the family. When maternal report and the booklet data were not in agreement, the health booklet data were preferentially selected. Breastfeeding duration was assessed by maternal report.

### 2.4. Statistical Analysis

We first present descriptive statistics, including the prevalence of overweight and obesity adjusted for complex sampling that used clustering design. We used ordinal regressive models with logit link adjusted to complex sample effects to determine the association of environmental, socioeconomic, and nutritional factors with overweight and obesity prevalence. We present minimally adjusted models that included covariates for child age and sex. Following the minimally adjusted, we took a multilevel hierarchical causal approach to multivariate analyses, in which all variables of each level (presented in each result table) were included in the initial model. To avoid adjusting for potential mediators, we did not adjust for pregnancy habits factors. All study data were double-entered twice using EpiInfo 2000 and tested for concordance. P-values lower than or equal to 0.05 were considered significant. Analysis was performed using SPSS Version 23 (SPSS Statistics for Windows, Version 23.0. IBM Inc., New York, NY, USA). 

### 2.5. Ethics

Written informed consent was obtained from participating women. Written consent for children was also given by mothers, and consent for adolescent minors was obtained from their parents or legal guardians. The PESMICs survey was approved by the Research Ethics Committee in Brazil, under the number 73516417.4.0000.5049. 

## 3. Results

PESMIC 2017 surveyed 3566 children from 20 municipalities in Ceará. This study analyzed 2059 children between 2 and 6 years of age from across the state. A summary of the study population characteristics is presented in Table 1. The mean age of the children in the sample was 46 ± 17 months, 50.4% of the population were males, with a general prevalence of overweight of 11.9% among boys and 12.1% among girls. Obesity was identified in 8.8% of boys and 7.1% of girls. Moreover, 58.1% of children were enrolled in the conditional cash transfer program (namely Bolsa Família in Brazil) and 59.1% of them presented with food insecurity. Of the mothers, 73.5% had up to 4 years of study and more than 80% of them did not work or worked at home. The percentage of children frequenting the nursery was 37.5%. Among the mothers, 4.8%, 13.2%, 5.7%, 53.5%, and 10.3% presented diabetes, hypertension, hypercholesterolemia, obesity, and depression, respectively. Percentage of children breastfed up to 16 months was 54.1%, and 44.1% of children were exclusively breastfed for up to 2 months. (Table 1). All other characteristics of these children are presented in Table 1.

The relationship of socioeconomic factors with child overweight and obesity is presented in Table 2. It was observed that the probability of childhood obesity was increasing as family income increases, with 5.7% in the first income quintile reaching 11% in the wealthiest quintile (adjusted hazard ratio (aHR) from first to last quintile 0.6 (95% CI 0.37–0.95), *p*-value = 0.03) This trend is observed in the same way when evaluating social groups that receive financial assistance from the government, with groups receiving aid (6.8%) and families who are eligible to receive, i.e., poorer (8.8%), have a probability of childhood obesity lower than the group that does not receive (9.8%), although without statistical significance in the multivariable analysis. Among the social strata, there was also a gradient of increased prevalence of childhood overweight with a higher social level. Families who had no food insecurity criteria had a higher probability of overweight (13.2%) and childhood obesity (8.8%) that families with some criterion of food insecurity. In families with fewer members, there is an increase of 55% in the risk of having overweight/obese children (aHR 1.55; 95% CI 1.02–2.36 *p*-value = 0.03).

The association of environmental factors with child overweight and obesity is presented in Table 3. Children who do not attend daycare had almost 30% higher chance of overweight and obesity (aHR 1.28; 95% CI 1–1.64; *p*_a_ value = 0.044). We also found that families with fewer children had more than 30% fewer overweight children (aHR 0.68; 95% CI 0.48–0.96), *p*_a_ value = 0.03. Female children of mothers who felt safe in the home environment were 40% less likely to be overweight (*p*_a_ value = 0.027).

The relationship of maternal comorbidities with overweight and obesity outcomes is presented in Table 4. We identified a higher risk for children of hypertense and obese mothers (aHR 1.97, 95% CI 1.22–3.16 and 1.48, 95% CI 1–2.2, respectively). We found no association between maternal diabetes and hypercholesteremia presentation and the outcome.

Children who breastfed during an intermediate time (between six and 16 months) were 1.44 more likely to be overweight than children who breastfed for a longer time (more than 16 months) (HRa 1.44; 95% CI 1.09–1.9), *p*_a_ = 0.01, as seen in Table 5. We found no association with breastfeeding in the first hour or taking vitamin A.

## 4. Discussion

We conducted a population-representative cross-sectional study of 3566 children aged 0–66 months of age in the state of Ceará, northeastern Brazil. We found that multiple environmental, socioeconomic, and nutritional factors were associated with child overweight and obesity. In terms of environmental and socioeconomic factors, lower income, overcrowded home, and attending daycare were associated with a lower prevalence of obesity. Maternal obesity and hypertension were identified as also linked to the outcome. Intermediate breastfeeding duration was associated with a higher prevalence of overweight and obesity, as compared to children breastfed for longer or shorter periods. 

In terms of socioeconomic factors, we found that lower income and lower social strata, as well as participating in the conditional cash transfer program was associated with a lower risk of obesity. Previous studies identified that the prevalence of obesity varies across settings, and different socioeconomic status (SES) groups are at different risks. For example, in China, the findings are similar to the ones of this study, but in the USA wealthier children are at a lower risk. [16] This different pattern may be due to the effect of actions in more developed countries, that slowed down the incidence of obesity in higher SES classes, as seen in England [17]. In Australia, from 1985 to 2015, the children with lower socioeconomic status (SES) had a greater chance of being in unhealthy weight categories [18]. In addition, in developed countries, where there is a low level of food insecurity in general, it is observed that low-income families have greater access to foods of low nutritional value, which are cheaper, and this has led the prevalence of obesity to increase in this group [19]. Our study contributes to the evidence of this association in a developing country of South America and the direction of the association. As food insecurity was not associated with obesity, we may think that lower SES groups may have lower access to specific types of foods, namely ultra-processed foods and sweetened beverages and worse feeding practices [20]. This may also justify that children attending daycare have a lower prevalence of obesity, as identified in our findings. In 2016, Brazil enacted the law called the canteen law (lei da cantina), which establishes criteria of nutritional quality for the diet used in daycare centers, which may also have contributed. As a result, ensuring education on better feeding practices focused in higher SES strata may provide improved nutritional outcomes.

In addition, we found that children who were born in families with more children in the house had a lower chance of being obese. Household crowding indicates the disorganization of the family practices and is linked to the density of residents in the home and, in Brazil, larger families are more common among the poorest. A recent systematic review has linked the higher degree of household disorganization within the home environment with obesity, probably by shaping children’s health behavior and leading to worst feeding practices [21]. Furthermore, crowding has been shown to be predictive of children’s outcomes above and beyond the influence of SES [22], as identified in the adjusted analysis of our study. Morover, children with siblings may be more propense to engage in physical activities, which may reduce the risk of obesity. Interventions to reduce the burden of disorganized households’ environment may have significant effects on nutritional outcomes.

We also found that maternal obesity and hypertension were associated with a higher prevalence of overweight and obesity in children, and this association was also identified in past studies in Brazil [23]. Additionally, other studies identified that after adjusting for all other factors, maternal obesity was the factor with a higher power of association with child obesity and this effect is not mediated by feeding practices, as maternal obesity has no significant influence on the feeding behavior of obese children [24,25]. This effect is also not due to maternal diabetes, as we see in our findings. In fact, this association may be explained by a direct child’s inheritance of genes that make the child susceptible to obesity, as energy balance seems to depend about 40% on genetic inheritance [26,27]. As a result, children who have obese and hypertense mothers may also be targeted for interventions aiming to diminish childhood obesity prevalence.

There is a robust literature linking breastfeeding with healthier weight categories [28,29,30], although the size of the effect of this relationship is often small [31,32]. This study adds to this evidence by identifying that the children with a higher prevalence of obesity are the ones with intermediate breastfeeding duration and this may be due to modification of the effect of other determinants by breastfeeding duration, as identified in other studies [23]. We hypothesize that early weaned children may receive increased parental and health services attention to feeding practices, as the children were not able to breastfeed and breastfeeding is consensual in Brazil now; on the other hand, children who breastfed during the suggested first six months of life, but then initiated another diet, may have a lower level of concern on their feeding behavior. As a result of these findings, more research is required on the relationship between breastfeeding initiation, duration, and complementary feeding on infant nutritional outcomes, particularly in low- and middle-income countries.

This study has a few limitations. First, the cross-sectional design of the study does not allow for analysis of child nutritional trajectories over time nor direct determination of causal relationships. Second, while the study was designed to be population representative of children in the State of Ceará, our findings may not be generalizable to children in other contexts.

## 5. Conclusions

Overall, we found that a range of environmental, socioeconomic, maternal, and breastfeeding factors were associated with nutritional outcomes among children of Ceará. As a result, integrated programs spanning from conception, or earlier, through the first years of life may reduce child obesity prevalence. This is one of the few studies that used populational representative samples in the developing world to study this association. Trials and studies of large-scale integrated interventions to address childhood obesity in Brazil at the population level are needed.

## Figures and Tables

**Table 1:** Prevalence of overweight and obesity in children aged 2–6 years among demographic and socioeconomic groups, and by environmental, family, and nutritional factors, Ceará, 2017.

			Overweight	Obesity
		n (%)	n (%; 95% CI)	n (%; 95% CI)
Prevalences			223 (12; 10.7–13.6)	148 (8; 6.7–9.5)
**Sample Characteristics**				
Age			46.64 ± 17.6 months	
Sex	Male	1039 (50.4)	110 (11.9; 9.7–14.4)	82 (8.8; 7–11)
	Female	1020 (49.5)	113 (12.1; 10.4–14.1)	66 (7.1; 5.6–8.9)
Mother’s ethnicity	White	336 (18.7)	40 (11.9; 8.7–16)	39 (11.6; 8.3–15.9)
	Brown	1323 (73.5)	164 (12.4; 10.7–14.3)	98 (7.4; 6–9.1)
	Black	137 (7.6)	13 (9.5; 5.8–15.1)	10 (7.3; 3.9–13.2)
	Other	5 (0.3)	–	–
**Socioeconomic Factors**				
Family income (quintiles)	1st	403 (20.1)	43 (11.8; 08.9–15.3)	21 (5.7; 3.7–8.6)
	2nd	427 (21.3)	45 (11.6; 8.6–15.6)	27 (7; 4.8–10)
	3rd	363 (18.1)	38 (11.4; 8.5–15.2)	29 (8.7; 6–12.4)
	4th	435 (21.7)	45 (11.5; 8.7–15)	32 (8.2; 5.9–11.2)
	5th	369 (18.4)	44 (13.4; 9.9–18)	36 (11; 7.9–15)
Social stratum (tercile)	1st	866 (42.8)	83 (10.5; 8.7–12.5)	55 (6.9; 5.2–9.1)
	2nd	512 (25.3)	55 (11.9; 8.9–15.7)	41 (8.9; 6.5–11.9)
	3rd	643 (31.8)	79 (13.9; 11.3–17)	49 (8.6; 6.5–11.2)
Receive government income support	Yes	1196 (58.1)	127 (11.7; 10.1–13.6)	74 (6.8; 5.3–8.8)
	Eligible	124 (6.0)	10 (8.8; 4.8–15.4)	10 (8.8; 4.7–15.7)
	No	740 (35.9)	86 (13.2; 10.8–15.9)	64 (9.8; 7.7–12.4)
Food	No	748 (40.8)	88 (13.2; 10.8–16)	59 (8.8; 6.7–11.5)
insecurity	Yes	1082 (59.1)	112 (11.4; 9.6–13.3)	72 (7.3; 5.7–9.3)
Number of	1st	646 (31.4)	75 (12.8; 10.3–15.8)	53 (9; 6.9–11.6)
persons in	2nd	1092 (53.2)	123 (12.5; 10.6–14.7)	73 (7.4; 5.9–9.3)
the house (tercile)	3rd	314 (15.3)	25 (8.8; 5.9–13)	21 (7.4; 4.9–11.1)
Medical appointment in the last three months	No	858 (42.3)	94 (12; 9.9–14.6)	68 (8.7; 6.8–11.1)
	Yes	1166 (57.6)	126 (12; 10.2–14.1)	80 (7.6; 6.2–9.3)
**Environmental Factors**				
Education level of household head	Fundamental	1408 (73.6)	162 (12.8; 11.1–14.7)	103 (8.1; 6.6–9.9)
	Medium	401 (20.9)	32 (8.7; 6.2–12.2)	23 (6.3; 4.1–9.4)
	Superior	102 (5.3)	13 (14.6; 8.8–23)	7 (7.8; 3.8–15.5)
Mother´s marital status	Alone	560 (27.9)	60 (11.7; 9.0–14.9)	43 (8.3; 6.1–11.4)
	With mate	1441 (72)	157 (12.1; 10.5–14)	103 (7.9; 6.5–9.7)
Mother works	Yes, out of home	383 (19.2)	51 (14.8; 11.4–19)	23 (6.7; 4.3–10.1)
	At home or does not work	1607 (80.7)	165 (11.3; 9.9–13)	120 (8.2; 6.7–10)
Child goes to day care	No	1283 (62.4)	144 (12.4; 10.7–14.3)	101 (8.7; 7.2–10.4)
	Yes	770 (37.5)	78 (11.3; 9–14)	47 (6.8; 5.1–9)
Median of children in the house	1	480 (23.3)	38 (8.9; 6.3–12.4)	27 (6.3; 4.2–9.3)
	2	1579 (76.6)	185 (12.9; 11.3–14.7)	121 (8.4; 7.1–10)
Mother feels safe from violence at home	No	242 (11.7)	19 (8.7; 5.7–13)	13 (5.9; 3.6–9.7)
	Yes	1754 (85.1)	198 (12.5; 11–14.1)	134 (8.4; 7.1–10)
**Biological Factors**				
Mother has diabetes	Yes	97 (4.8)	15 (17.6; 10.7–27.5)	7 (8.2; 3.6–17.4)
	No	1904 (95.1)	203 (11.8; 10.4–13.3)	140 (8.1; 6.8–9.6)
Mother has hypertension	Yes	266 (13.2)	38 (15.8; 11.8–20.8)	24 (10; 6.6–14.8)
	No	1735 (86.7)	179 (11.4; 9.9–13.2)	122 (7.8; 6.4–9.4)
Mother has hypercholesterolemia	Yes	113 (5.7)	13 (12.7; 7.7–20.2)	12 (11.7; 7.2–18.4)
	No	1865 (94.2)	201 (11.9; 10.4–13.6)	132 (7.8; 6.5–9.3)
Maternal obesity	Yes	1007 (53.5)	121 (13.4; 11.2–15.9)	84 (9.3; 7.4–11.5)
	No	873 (46.4)	82 (10.3; 8.3–12.6)	54 (6.8; 5.2–8.7)
Maternal depression	Yes	202 (10.3)	20 (10.9; 7.2–16.2)	12 (6.6; 3.7–11.3)
	No	1754 (89.7)	194 (12.3; 10.8–13.9)	134 (8.5; 7.1–10.1)
Type of delivery	Normal	453 (41.7)	35 (8.5; 6.2–11.6)	29 (7.1; 4.9–10.1)
	Cesarean	631 (58.2)	66 (11.7; 9.4–14.4)	37 (6.5; 4.5–9.4)
Macrosomia	No	1755 (94.3)	181 (11.4; 10–13.1)	129 (8.1; 6.8–9.7)
	Yes	105 (5.6)	20 (20; 13.5–28.4)	5 (5; 2.1–11.2)
**Nutritional Factors**				
Breastfeeding time	Up to 5 months	499 (26)	66 (15.1; 11.9–18.9)	26 (5.9; 3.9–8.9)
	6–16 months	541 (28.1)	59 (12; 9.1–15.7)	56 (11.4; 8.8–14.7)
	More than 16 months	879 (45.8)	85 (10.5; 8.8–12.6)	57 (7.1; 5.4–9.2)
Exclusive breastfeeding time	Up to 2 months	906 (44.1)	107 (13; 11–15.3)	71 (8.6; 6.8–10.8)
	Up to 4 months	419 (20.4)	44 (12; 9.2–15.5)	23 (6.3; 4–9.7)
	Up to 6 months	726 (35.3)	71 (10.8; 8.6–13.4)	54 (8.2; 6.2–10.7)
Child breastfed in first hour of life	No	415 (20.2)	45 (12.1; 9.2–15.8)	32 (8.6; 6.2–11.9)
	Yes	1639 (79.8)	177 (12.0; 10.4–13.8)	116 (7.9; 6.4–9.6)
Child took vitamin A	Yes	1757 (85.2)	196 (12.3; 10.9–14.0)	128 (8; 6.7–9.6)
	No	303 (14.7)	27 (09.9; 06.9–14.0)	20 (7.3; 4.6–11.4)

**Table 2:** Socioeconomic factors associated with overweight/obesity in children aged 2–6 years, Ceará, 2017.

		Childhood Overweight and Obesity				
		n (%)	_m_HR (95% CI)	*p* Value	_a_HR (95% CI)	*p*_a_ Value
Family income (quintiles)	1st	64 (17.5)	0.61 (0.43–0.86)	**0.006**	0.6 (0.37–0.95)	**0.031**
	2nd	72 (18.7)	0.67 (0.46–0.96)	**0.033**	0.63 (0.39–1.01)	0.057
	3rd	67 (20.1)	0.76 (0.52–1.13)	0.183	0.71 (0.46–1.1)	0.129
	4th	77 (19.7)	0.74 (0.52–1.03)	0.082	0.71 (0.48–1.04)	0.086
	5th	80 (24.5)	1		1	
Social stratum (terciles)	1st	138 (17.4)	0.74 (0.57–0.97)	**0.033**	0.92 (0.66–1.28)	0.622
	2nd	96 (20.8)	0.92 (0.67–1.27)	0.642	1.07 (0.76–1.5)	0.678
	3rd	128 (22.6)	1		1	
Receive government income support	Yes	201 (18.5)	0.74 (0.57–0.95)	**0.021**	0.9 (0.65–1.24)	0.535
	Eligible	20 (17.5)	0.68 (0.39–1.19)	0.185	0.72 (0.38–1.36)	0.320
	No	150 (22.9)	1		1	
Food	No	147 (22)	1.21 (0.95–1.54)	0.120	1.08 (0.83–1.4)	0.558
insecurity	Yes	184 (18.7)	1		1	
Number of	1st	128 (21.9)	1.42 (1.01–2)	**0.041**	1.55 (1.02–2.36)	**0.038**
persons in	2nd	196 (20)	1.23 (0.87–1.75)	0.231	1.18 (0.79–1.75)	0.409
the house (tercile)	3rd	46 (16.3)	1		1	

_m_HR—minimally adjusted hazard ratio: _a_HR–adjusted hazard ratio; *p*_a_ value—adjusted *p*-value. Bold values are significant *p* values.

**Table 3:** Home environment factors associated with overweight/obesity in children aged 2–6 years, Ceará, 2017.

		Childhood Overweight and Obesity				
		n (%)	_m_HR (95% CI)	*p* Value	_a_HR (95% CI)	*p*_a_ Value
Education level of household head	Fundamental	265 (20.9)	0.84 (0.5–1.4)	0.516	0.8 (0.48–1.32)	0.805
	Medium	55 (15)	0.57 (0.32–1.02)	0.059	0.57 (0.32–1.01)	0.574
	Superior	20 (22.4)	1		1	
Mother´s marital status	Alone	103 (20.1)	1 (0.76–1.3)	0.996	1 (0.75–1.33)	0.993
	With mate	260 (20.1)	1		1	
Mother works	Yes, out of home	74 (21.5)	1.05 (0.8–1.38)	0.692	1.01 (0.75–1.35)	0.934
	At home or does not work	285 (19.6)	1		1	
Child goes to day care	Yes	245 (21.1)	1.31 (1.04–1.64)	**0.019**	1.28 (1–1.64)	**0.044**
	No	125 (18.1)	1		1	
Median of children in the house	1	65 (15.3)	0.64 (0.46–0.88)	**0.006**	0.68 (0.48–0.96)	**0.030**
	2	306 (21.4)	1		1	
Mother feels safe at home	No	332 (21)	0.63 (0.43–0.93)	**0.020**	0.6 (0.39–0.94)	**0.027**
	Yes	32 (14.7)	1		1	

_m_HR—minimally adjusted hazard ratio; _a_HR—adjusted hazard ratio; *p*_a_ value—adjusted *p*-value. Bold values are significant *p* values.

**Table 4:** Maternal comorbidities and gestational factors associated with overweight/obesity in children aged 2–6 years, Ceará, 2017.

		Childhood Overweight and Obesity				
		n (%)	_m_HR (95% CI)	*p* Value	_a_HR (95% CI)	*p*_a_ Value
Mother has diabetes	Yes	22 (25.8)	1.32 (0.84–2.09)	0.223	0.89 (0.46–1.72)	0.737
	No	343 (19.9)	1		1	
Mother has hypertension	Yes	62 (25.8)	1.42 (1.03–1.95)	**0.022**	1.97 (1.22–3.16)	**0.005**
	No	301 (19.2)	1		1	
Mother has hypercholesterolemia	Yes	25 (24.5)	1.28 (0.85–1.94)	0.231	1.47 (0.66–3.26)	0.342
	No	333 (19.8)	1		1	
Maternal depression	Yes	32 (17.4)	0.76 (0.53–1.09)	0.146	0.55 (0.27–1.11)	0.099
	No	328 (20.7)	1		1	
Maternal obesity	Yes	205 (22.7)	1.36 (1.08–1.71)	**0.009**	1.48 (1–2.2)	**0.050**
	No	136 (17.1)	1		1	
Type of delivery	Normal	64 (15.6)	0.84 (0.6–1.19)	0.353	0.94 (0.65–1.36)	0.755
	Cesarean	103 (18.2)	1		1	
Macrosomia	No	310 (19.6)	0.81 (0.53–1.22)	0.332	0.77 (0.37–1.6)	0.491
	Yes	25 (25)	1		1	

_m_HR—minimally adjusted hazard ratio; _a_HR—adjusted hazard ratio; *p*_a_ value—adjusted *p*-value. Bold values are significant *p* values.

**Table 5:** Nutritional factors associated with overweight/obesity in children aged 2–6 years, Ceará, 2017.

		Childhood Overweight and Obesity				
		n (%)	_m_HR (95% CI)	*p* Value	_a_HR (95% CI)	*p*_a_ Value
Breastfeeding time	Up to 5 months	92 (21.1)	1.18 (0.89–1.56)	0.229	1.14 (0.85–1.54)	0.367
	Up to 16 months	115 (23.5)	1.44 (1.09–1.89)	**0.010**	1.44 (1.09–1.9)	**0.010**
	More than 16	142 (17.7)	1		1	
Exclusive breastfeeding time	Up to 2 months	178 (21.6)	1.17 (0.92–1.49)	0.195	1.17 (0.89–1.54)	0.239
	Up to 4 months	67 (18.3)	0.96 (0.69–1.34)	0.829	0.97 (0.68–1.38)	0.872
	Up to 6 months	125 (19)	1		1	
Child breastfed in first hour of life	No	77 (20.7)	1.05 (0.79–1.39)	0.707	1.19 (0.87–1.62)	0.264
	Yes	293 (19.8)	1		1	
Child took vitamin A	Yes	324 (20.4)	1.17 (0.84–1.62)	0.339	1.16 (0.8–1.67)	0.420
	No	47 (17.3)	1		1	

_m_HR—minimally adjusted hazard ratio; _a_HR—adjusted hazard ratio; *p*_a_ value—adjusted *p*-value. Bold values are significant *p* values.
